# A 21.6 kDa tegumental protein of *Clonorchis sinensis* induces a Th1/Th2 mixed immune response in mice

**DOI:** 10.1002/iid3.235

**Published:** 2018-10-09

**Authors:** EunJoo Chung, Yu Jung Kim, Myoung‐Ro Lee, Shin‐Hyeong Cho, Jung‐Won Ju

**Affiliations:** ^1^ Division of Vectors and Parasitic Diseases, Center for Laboratory Control of Infectious Diseases Korea Centers for Disease Control & Prevention Osong 28159 Republic of Korea

**Keywords:** *Clonorchis sinensis*, dendritic cells, T lymphocytes

## Abstract

**Introduction:**

*Clonorchis sinensis* is a major parasite affecting the Korea population. Despite the high infection rate and pathogenicity, very few studies have been conducted to investigate the immune responses against the proteins of *C. sinensis*.

**Methods:**

In this study, in vitro immune response induced by a recombinant 21.6 kDa tegumental protein derived from *C. sinensis* (rCsTegu21.6) was confirmed in murine dendritic cells and T cells. For the in vivo analysis, each mouse was immunized three times. Total serum IgG and T cell cytokine production were determined by ELISA, while T cell proliferation was detected by a WST (Water‐Soluble Tetrazolium salt)‐1 assay.

**Results:**

In vitro tests indicated that rCsTegu21.6 treatment increased the expression of surface molecules, such as CD40 (77%), CD80 (52%) and CD86 (46%), on murine dendritic cells and the secretion of cytokines (TNF‐α, IL‐6, IL‐1β, IL‐10, and IL‐12p70). Moreover, co‐culturing dendritic cells activated by rCsTegu21.6 with allogenic T cells induced T cell proliferation over time. rCsTegu21.6 also stimulated specific antibody production and cytokine secretion [IL‐2, IL‐4, and interferon (IFN)‐γ)] from T cells following immunization in vivo. Notably, rCsTegu21.6 predominantly induced IgG1 production and secretion of the Th2 cytokine IL‐4, regardless of the type of adjuvant used.

**Conclusion:**

These results serve as a foundation for the development of tegumental protein‐based vaccines against *C. sinensis*.

## Introduction

The liver fluke *Clonorchis sinensis* is a well‐known fish‐borne trematode that is prevalent in many Asian countries, including South Korea, China, northern Vietnam, and far‐eastern Russia 1. Adult *C. sinensis* can survive in the bile ducts for an extended period, leading to the development of various diseases, including jaundice, indigestion, biliary inflammation, bile duct obstruction, cholelithiasis, cholestasis, cholangitis, cholecystitis, liver cirrhosis, and most severely, bile duct cancer or cholangiocarcinoma (CCA) 1, 2, 3. Because of these risks, *C. sinensis* was classified as a probable carcinogen (group 2A) in 1994 by the International Agency for Research on Cancer (IARC) of the World Health Organization (WHO). However, in 2009, following additional supplemental studies, *C. sinensis* was reclassified as a group 1 carcinogen 4, 5.

In South Korea, most parasites, including soil‐transmitted parasites as well as those causing filariasis, are highly controlled, but *C. sinensis* has remained prevalent because of the common practice of eating raw freshwater fish 6, 7. In fact, in a 2008 case‐control study in Korea, radiological evidence of *C. sinensis* and recent consumption of raw freshwater fish were significantly correlated with gallstone formation 8. An etiological role for *C. sinensis* was also previously suggested following a study demonstrating a correlation between infection and both cholangiocarcinoma as well as clonorchiasis 9.

Despite these risks, there is currently no commercially available vaccine for *C. sinensis* infection. However, a number of researchers are currently investigating drug targets to prevent *C. sinensis* infection. These targets include excretory/secretory products 10, tegumental proteins 11, 12, and metabolism‐related enzymes 13, 14, 15, 16, 17. Moreover, proteins that have been identified as effective vaccine targets for other parasites are also being evaluated for their use in *C. sinensis* vaccine development 18.

Notably, the tegument of blood or liver flukes is generally considered the most susceptible target for vaccines and drugs because it is a primary site of interaction between the host and parasite, in addition to being responsible for both the host immune response and survival of the parasite 19, 20, 21. Although the effects of vaccines produced against tegumental proteins from *Schistosoma sp*. and *Fasciola sp*. have been previously studied 19, 20, only a few tegument proteins from *C. sinensis* have been characterized as vaccine candidates. Among these, protective effects and immunogenicity have been confirmed for vaccines against CsPmy 11 and CsTP22.3 12, while only immunolocalization and antibody characterization have been conducted for the TP20.8 22, 23 and TP31.8 24 vaccines. Therefore, additional work is needed in order to develop novel vaccines to prevent *C. sinensis* infection.

In this study, we have characterized the CsTegu21.6 protein sequence, using previously documented bioinformatic analysis protocols 25. Furthermore, the immunological potential of the CsTegu21.6 protein as an inducer of the host's immune response to *C. sinensis* was assessed. To our knowledge, this is the first study investigating the CsTegu21.6 tegumental protein for use as a *C. sinensis* vaccine.

## Materials and Methods

### Preparation and visualization of rCsTegu21.6

CsTegu21.6 protein expression was carried out following a previously published protocol 25. Briefly, clones of cDNA coding the CsTegu21.6 protein were obtained from a *C. sinensis*c DNA library. The cDNA was amplified using forward (5′‐GGG CAA GGT ACC ATG GAG CCA TTC TTA GAA G‐3′) and reverse (5′‐CCC GTT AAG CTT TCA GCT TGG TGT CTT CCA C‐3′) primers. The PCR product was then cloned into the pRSET vector (Novagen, Madison, WI), and recombinant plasmids were used to transform *Escherichia coli* BL21 (DE3) pLysS cells (Invitrogen, Carlsbad, CA). The recombinant CsTegu21.6 protein was produced in LB media containing ampicillin and induced by adding 0.5 mM isopropyl‐B‐d‐thiogalactopyranoside (IPTG) for 20 h at 16°C. Cells were harvested by centrifugation and resuspended in buffer containing 50 mM NaH_2_PO_4_ and 300 mM NaCl (pH 8.0). Subsequently, the cell suspension was sonicated on ice, and the supernatant was collected by centrifugation at 13,000*g* for 30 min.

His‐tagged recombinant protein was purified using a Protino® Ni‐IDA (Ni^2+^‐iminodiacetic acid) column (Macherey‐Nagel, Bethlehem, PA) under native conditions. First, the column was equilibrated with Lysis‐Equilibration‐Wash (LEW) buffer (50 mM NaH_2_PO_4_, 300 mM NaCl [pH 8.0]), and then the protein solution was loaded onto the equilibrated column. For endotoxin reduction, a washing procedure was performed according to previous reports 26, 27. The column was first washed with 50 column volumes of 0.1% Triton X–114 (Sigma–Aldrich, St. Louis, MO) in LEW buffer, followed by a second wash with 20 column volumes of the same buffer without Triton X–114 under cold conditions. Finally, rCsTegu21.6 protein was eluted with elution buffer (50 mM NaH_2_PO_4_, 300 mM NaCl, 250 mM imidazole [pH 8.0]). The purified proteins were then concentrated, and the buffer was exchanged for Dulbecco's phosphate‐buffered saline (DPBS; pH 7.4) using Amicon Ultra‐4 centrifugal filter devices (Millipore, Billerica, MA). The protein concentration was quantified by Bradford assay.

To completely remove residual endotoxins from the rCsTegu21.6 protein, an additional endotoxin removal step was performed using Detoxi‐Gel endotoxin removal columns (Pierce, Rockford, IL). The endotoxin level in recombinant proteins was measured using the Limulus Amoebocyte Lysate (LAL) Chromogenic Endotoxin Quantitation kit (Pierce), and was found to be negligible (<1 EU/mL). In addition, polymyxin B (Sigma–Aldrich) was used to pre‐treat the rCsTegu21.6 protein before use in the immune response analysis.

The rCsTegu21.6 protein was analyzed by 12% sodium dodecyl sulfate polyacrylamide gel electrophoresis (SDS–PAGE) and then transferred onto PVDF membranes (Millipore). The membranes were then stained with horseradish peroxidase (HRP)‐conjugated anti‐His IgG (Abcam, Cambridge, UK). The protein was detected using ECL western blotting detection reagent (GenDEPOT, Barker, TX) and visualized using a ChemiDoc imaging system (BioRad, Hercules, CA).

### Production of anti‐rCsTegu21.6 antibodies and immunolocalization

To produce anti‐rCsTegu21.6 antibodies, 50 µg/100 µL of each protein was emulsified with an equal volume of Freund's complete adjuvant and injected into BALB/c mice. After 2 weeks, a boost immunization was performed by again injecting 50 µg/100 µL of protein with 100 µL Freund's incomplete adjuvant. This process was also repeated after an additional 2 weeks. Totally, immunization was conducted three times. Anti‐sera were obtained 2 weeks after the final injection, and the specific binding of the anti‐sera was tested by immunoblotting and enzyme‐linked immunosorbent assay (ELISA; data not shown).

To determine the protein localization of rCsTegu21.6 in the tegument of *C. sinensis*, immunohistochemical analysis was conducted using the purified mouse anti‐rCsTegu21.6 antibodies. Parasite‐infected rabbit liver tissue (including the worms in the biliary tract) was isolated and fixed in 4% paraformaldehyde. The tissue was then dehydrated using a graded ethanol series and embedded in paraffin blocks. Sections (4‐µm thick) were mounted on glass slides, deparaffinized, rehydrated, and washed with distilled water. Following antigen retrieval and blocking, the slides were incubated with rCsTegu21.6 or non‐immunized mouse sera diluted 1:100 in PBS at room temperature for 2 h, and washed two times with PBS. After incubation with DAKO Envision kit detection system reagent (Agilent, Santa Clara, CA) for 30 min, the slides were washed two times in PBS and then rehydrated. The stained tissue sections were observed under a light microscope (Olympus, Tokyo, Japan).

### Mouse bone marrow‐derived dendritic cell (mBMDC) activation by rCsTegu21.6

The femurs and tibiae of C57BL/6 mice were removed with scissors and washed with DPBS. Bone marrow was extracted from the bones with both ends cut off. After washing with DPBS, red blood cells were removed by adding 1 mL of red blood cell lysis buffer (Sigma–Aldrich). The isolated bone marrow cells were then suspended in pre‐warmed culture medium (20 ng/ml granulocyte–macrophage colony‐stimulating factor [GM‐CSF], 20 ng/mL interleukin [IL]‐4 [Peprotech, Rocky Hill, NJ, USA], 10% fetal bovine serum [FBS; Gibco, Carlsbad, CA], 1% penicillin‐streptomycin in RPMI‐1640 media [Gibco]) and cultured in 6‐well plates at a concentration of 2 × 10^6^ cells/mL. The culture medium was replaced every 3 days for 8 days. Cells were then centrifuged at 2000 *rpm* for 5 min and resuspended in pre‐warmed culture medium. Finally, cells were seeded in a 6‐well plate at a density of 2 × 10^6^ cells/well. rCsTegu21.6 protein was pre‐incubated with 50 µg/mL polymyxin B for 1 h at 4°C. Then, varying concentrations of rCsTegu21.6 (1, 5, 10 µg/mL) or 100 ng/mL lipopolysaccharide (LPS; Sigma–Aldrich) were used to treat the mBMDCs. The treated cells were incubated for 18 h in a humidified 5% CO_2_ incubator at 37°C.

After harvesting the cells, the supernatant was collected and used for cytokine quantification. The concentration of cytokines (IL‐6, IL‐1β, IL‐12p70, IL‐10, and tumor necrosis factor [TNF]‐α) was measured by ELISA using kits specific for each (R&D Systems, Minneapolis, MN), according to the manufacturer's instructions.

Harvested cells were also stained with fluorescein isothiocyanate (FITC)‐conjugated anti‐mouse/cluster of differentiation (CD) 11c antibody along with phycoerythrin (PE)‐conjugated anti‐mouse CD40, CD80, and CD86 antibodies (all from eBioscience, San Diego, CA) for 30 min at 4°C. After staining, the cells were washed twice with DPBS and resuspended in 4% paraformaldehyde. The fluorescence intensity was detected using a FACSVerse flow cytometer (BD Biosciences, Franklin Lakes, NJ) and analyzed using FlowJo software (Ashland, OR).

### Intracellular cytokine production test

Cells were processed with a Cytofix/Cytoperm kit (BD Biosciences), according to the manufacturer's instructions. Briefly, rCsTegu21.6‐treated mBMDCs (1 × 10^6^ cells/mL) were harvested and the supernatant was removed. The pellet was then washed with staining buffer (2.5% FBS in DPBS) and incubated with 1 µg/mL of FITC‐conjugated anti‐mouse CD11c IgG (eBioscience) at 4°C for 30 min. After washing with staining buffer, the cell pellet was collected by centrifugation and permeated using Cytofix/Cytoperm solution (BD Biosciences). The permeabilized cells were washed twice in 1× Perm/Wash solution (BD Biosciences). To stain the intracellular cytokines, the cells were incubated in 1 µg/mL PE‐conjugated anti‐mouse IL‐10, IL‐12, or interferon (IFN)‐γ IgG (eBioscience) at 4°C for 30 min under protective light conditions. Stained cells were then washed twice in 1× Perm/Wash solution and stored in 4% paraformaldehyde solution at 4°C until use. The fluorescence intensity of the intracellular cytokines was measured by flow cytometry, as described above.

### Analysis of the T cell response in rCsTegu21.6‐treated mBMDCs

To prepare allogenic T cells, splenocytes were obtained by grinding a spleen of an 8‐week‐old BALB/c mouse. Isolation of CD3^+^ T cells was performed via negative selection using a Pan T cell isolation kit (MiltenyiBiotec, BergischGladbach, Germany). The carboxyfluorescein succinimidyl ester (CFSE), a dye used to stain the membrane, was attached to the T cells, using a Cell Trace CFSE Cell Proliferation kit (Molecular Probes, Eugene, OR). mBMDCs were seeded onto a 6‐well at a density of 2 × 10^6^ cells/well and stimulated with 5 µg/mL rCsTegu21.6 protein for 18 h. After washing, 5 × 10^4^ mBMDCs were seeded again in a 96‐well round‐bottom plate. The plate was then incubated with CFSE‐labeled T cells (5 × 10^5^ cells/well) in a humidified 5% CO_2_ incubator at 37°C.

Cells were harvested at 4, 5, and 6 days after seeding, and the supernatants were stored at −20°C until testing. The harvested cells were washed two times and labeled with 1 µg/mL PE‐conjugated anti‐mouse CD3e antibody (eBioscience) at 4°C for 30 min. The labeled cells were then washed twice with DPBS and resuspended in 4% paraformaldehyde. The fluorescence intensity was detected using a FACSVerse flow cytometer (BD Biosciences) and analyzed with FlowJo software (Ashland). The level of cytokines (IL‐2, IL‐4, and IFN‐γ) in the T cell supernatant was measured by ELISA kits (R&D Systems) according to the manufacturer's instructions.

### Immunization with rCsTegu21.6 and sample collection

rCsTegu21.6 immunization was performed using six groups of C57BL/6 mice (*n* = 5 per group):group 1 (rCsTegu21.6 protein solution without adjuvant), group 2 (rCsTegu21.6 protein mixed with alum adjuvant), group 3 (rCsTegu21.6 protein emulsified with Freund's adjuvant [FA]), group 4 (PBS alone, control); group 5 (PBS mixed with alum adjuvant, control), and group 6 (PBS mixed with FA, control). Each mouse was injected intraperitoneally with 50 µg/200 µL of the designated mixtures. First and second booster injections were conducted on days 14 and 28 after initial injection using 50 µg/200 µL of rCsTegu21.6 protein (or PBS for the controls) emulsified in incomplete Freund's or alum adjuvant. Mice were sacrificed 7 days following the final immunization with CO_2_ inhalation followed by heart puncture. The serum was collected and centrifuged at 5000 *rpm* for 3 min and stored at −20°C until analysis. To detect the cytokine levels in the T cells of these mice, their spleens were removed from under the left rib, using scissors.

### Quantification of antibodies against rCsTegu21.6

To quantitate the antibodies produced in the immunized mice, a 96‐well plate was coated with 5 µg/mL rCsTegu21.6 protein for 18 h at 4°C. After three washes with PBS containing 0.05% Tween20 (PBST), the protein‐coated plate was blocked with PBST containing 5% skim milk at 37°C for 1 h. After additional washing, the plate was incubated with mouse serum from each group diluted in blocking buffer at 37°C for 1 h. The plate was then washed, and 100 µL of HRP‐conjugated rabbit anti‐mouse IgG (1:10,000 dilution; Sigma–Aldrich) was added. After incubating for 2 h, 100 µL of TMB substrate solution (GenDEPOT) was added to each well and incubated for an additional 30 min at room temperature. This reaction was ended by adding 100 µL of TMB Stop Buffer (GenDEPOT) to the wells. The optical density was immediately detected using a microplate reader at 450 nm (reference wavelength: 620 nm). To identify the IgG subtypes, antibody isotyping was performed using HRP‐conjugated goat anti‐mouse IgG1 and IgG2a (BioRad, Hercules, CA).

### Measurement of rCsTegu21.6‐induced cell proliferation and cytokine secretion

After grinding of the isolated mouse spleen, the T cells were purified from the splenocytes using a Pan T cell isolation kit (MiltenyiBiotec). Isolated T cells were then suspended in cell culture medium (RPMI 1640 with 10% FBS and 1% antibiotics) and plated in a 96‐well plate at a density of 1 × 10^5^ cells/well in a final volume of 100 µL/well. These plates were then incubated with or without rCsTegu21.6 protein (10 µg/mL) at 37°C in a 5% CO_2_ incubator for 70 h. To evaluate changes in proliferation, 10 µL of WST‐1/electro coupling solution (BioVision, Milpitas, CA) was added to each well and incubated under standard culture conditions for 4 h. The absorbance was detected using a microplate reader at 450 nm (reference wavelength: 620 nm). Additionally, T cells were also harvested after the 70 h incubation period, and their supernatant was collected for cytokine detection. The levels of IL‐2, IL‐4, and IFN‐γ were detected with ELISA kits (R&D Systems).

### Statistical analysis

Results are expressed as the mean ± standard error (SEM). The statistical significance of the observed differences was assessed by student *t*‐test with the acceptable *P*‐value set at less than 0.05. Statistical data were analyzed using GraphPad Prism version 5.01 software (GraphPad Software, San Diego, CA).

## Results

### Identification, expression, and immunolocalization of rCsTegu21.6

**Figure 1:**
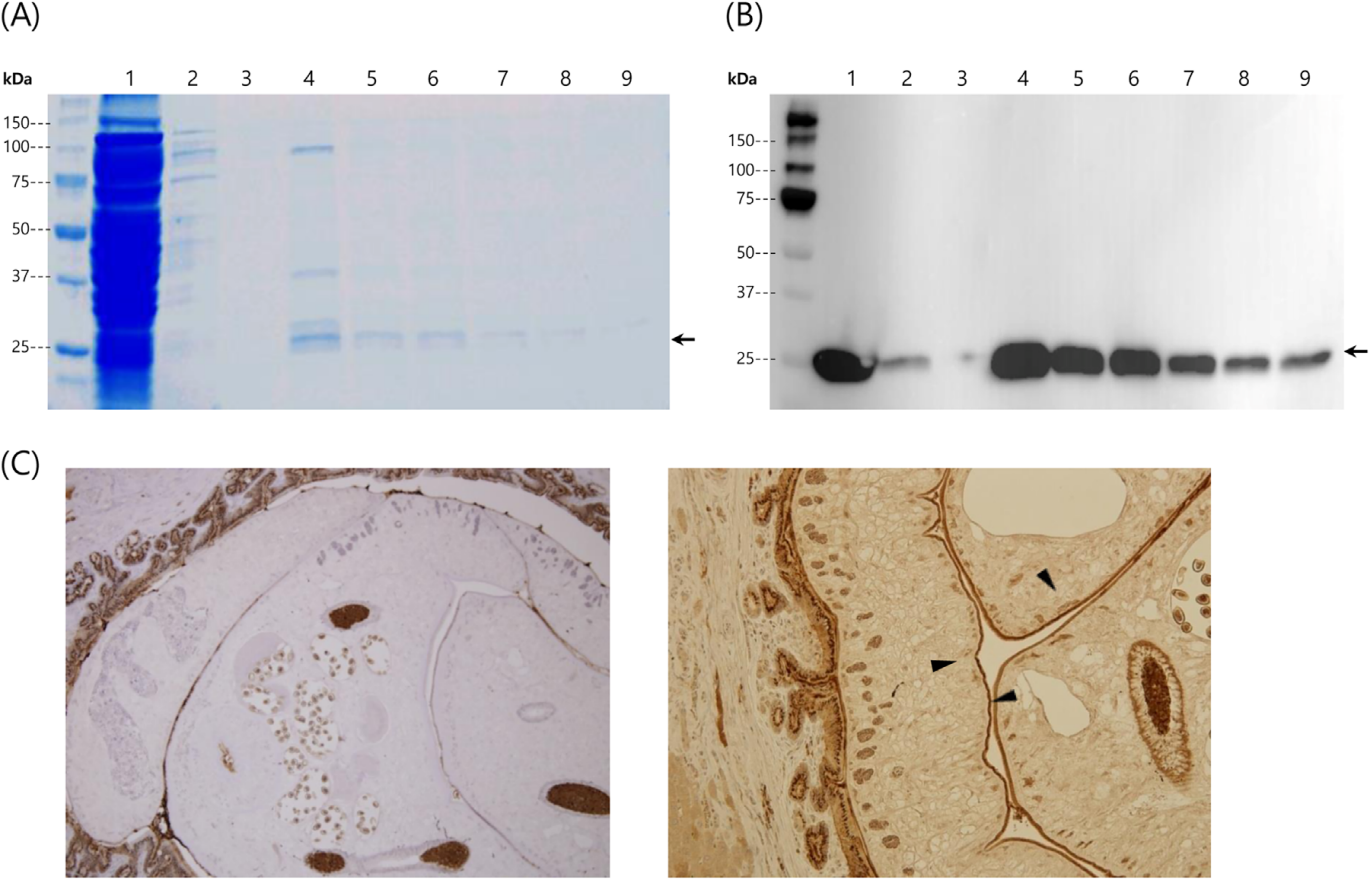
Expression and identification of recombinant CsTegu21.6 protein. (A) SDS–PAGE analysis of rCsTegu21.6 purified using a Ni‐IDA column (1, flow‐through; 2–3, washing; 4–9, elution). (B) Western blot image is showing the purified rCsTegu21.6 protein using anti‐His antibodies (1, flow‐through; 2–3, washing; 4–9, elution). (C) Immunolocalization of rCsTegu21.6 in the tegument of adult *C. sinensis* worms present in infected rabbit liver tissue sections, using CsTegu21.6‐immunized mouse sera. Sera from naïve mouse (*left panel*) were used as the negative control. CsTegu21.6 localization is indicated by arrows (*right panel*). CsTegu21.6 is mainly located in the tegument of adult *C. sinensis*.

The full protein sequence of CsTegu21.6 was obtained from GenBank (accession number AEI69651.1). The CsTegu21.6 protein is 188 amino acids with a putative molecular weight of 21.6 kDa, while the recombinant CsTegu21.6 protein is predicted to be 24.19 kDa because the 6 × His tag was added for the affinity purification of recombinant protein. rCsTegu21.6 protein was expressed with a 6 × His tag in BL21 (DE3) *E. coli* upon induction of IPTG (Figure 1A), and protein bands were identified with anti‐His antibodies by Western blotting (Figure 1B).

In the immunohistochemical and localization analyses (performed using mouse anti‐rCsTegu21.6 sera), CsTegu21.6 was mainly located in the tegument of adult *C. sinensis* (Figure 1C) and was not observed in other tissues. Furthermore, CsTegu21.6 not detected in sections incubated with either pre‐immune mouse sera.

### Surface marker expression and cytokine production in mBMDCs treated with rCsTegu21.6

**Figure 2:**
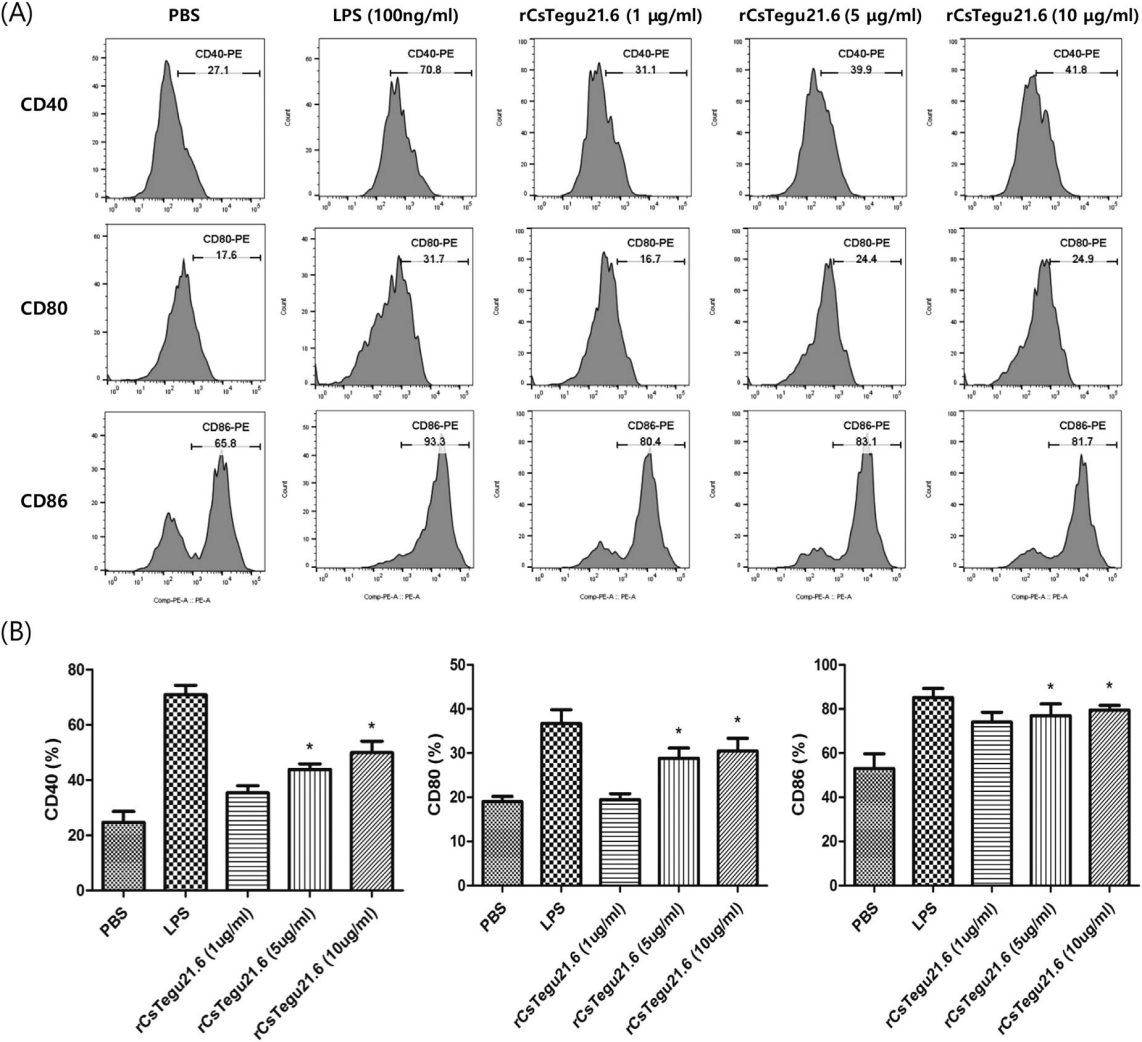
rCsTegu21.6 treatment induced surface marker expression on mBMDCs in a dose‐dependent manner. (A) mBMDCs were isolated from C57BL/6 mice and stimulated with LPS (100 ng/mL) or rCsTegu21.6 (1, 5, or 10 µg/mL) for 18 h. Harvested cells were labeled with FITC‐conjugated anti‐mouse CD11c and PE‐conjugated anti‐mouse CD40, CD80, and CD86 antibodies, individually. Fluorescence intensity was measured by flow cytometry. (B) Statistical results of the surface marker expression on mBMDCs. Data are shown as the mean ± standard error (SEM) and are representative of three independent experiments. *P < 0.05 compared to the PBS‐treated control group.

Dendritic cells are antigen‐presenting cells that can be stimulated to express immune response‐inducing surface molecules, such as CD40, CD80 and CD86, by various molecules. Here, rCsTegu21.6 was observed to induce the expression of these surface markers in mBMDCs in a dose‐dependent manner at a concentration of 5 µg/mL or more (Figure 2).

**Figure 3:**
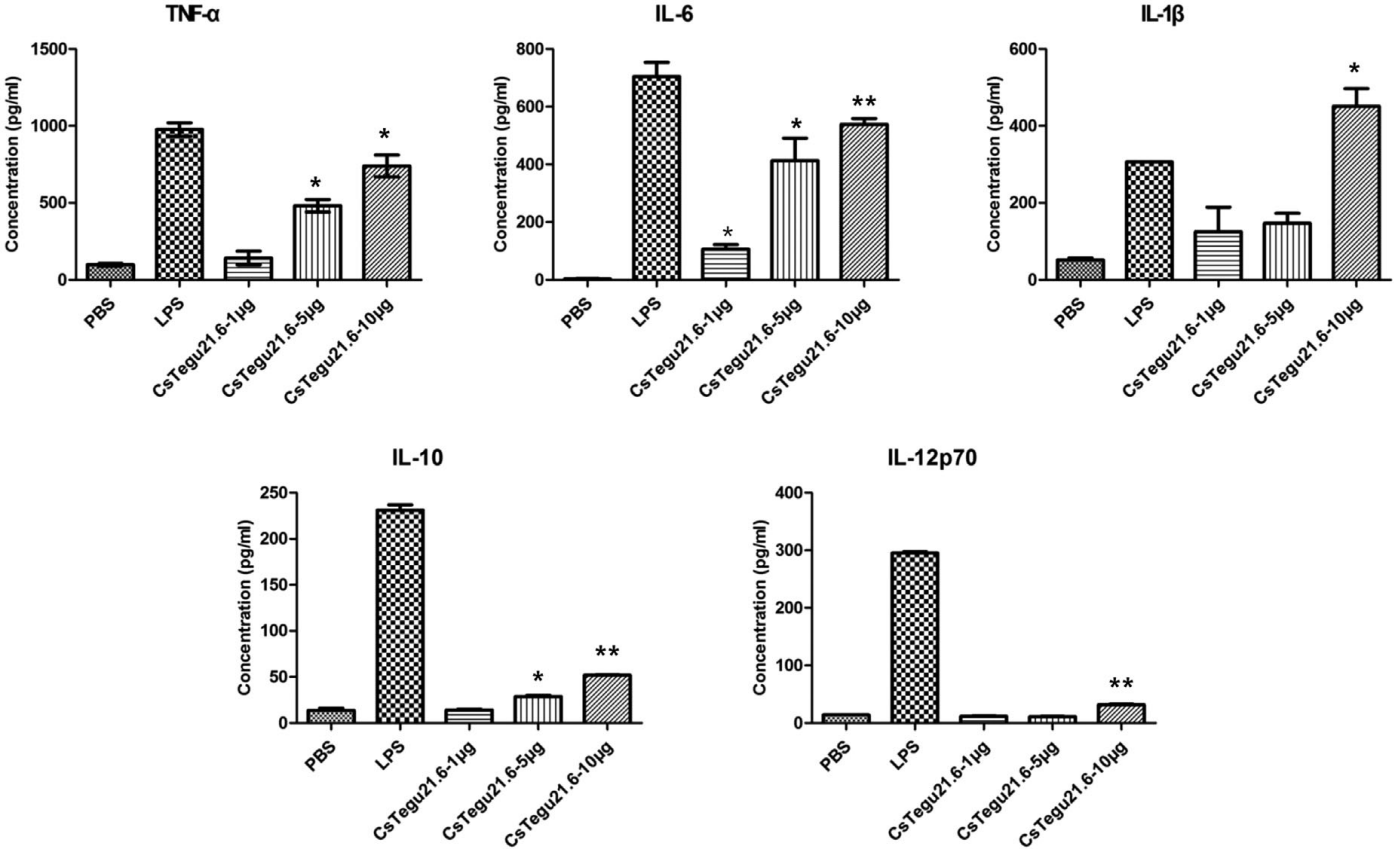
Cytokine secretion from rCsTegu21.6‐treated mBMDCs increased in a dose‐dependent manner. mBMDCs from C57BL/6 mice were activated with LPS or rCsTegu21.6 (1, 5, or 10 µg/mL) for 18 h. The concentration of secreted cytokines in the supernatant was quantified by ELISA. The results are shown as the mean ± standard error (SEM) and are representative of two independent experiments. *P < 0.05, **P < 0.01 compared to the PBS‐treated control group.

**Figure 4:**
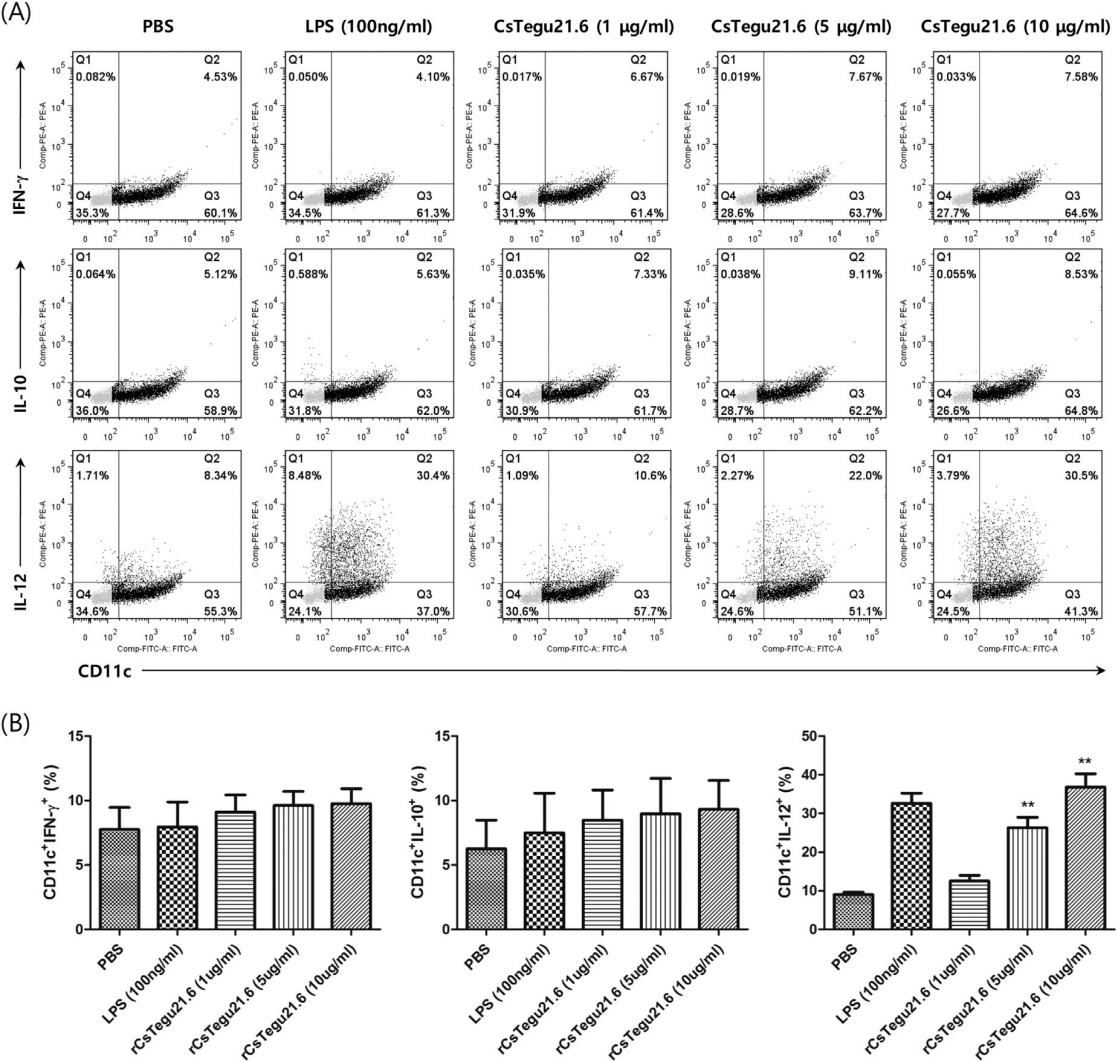
Production of intracellular IL‐12, but not IL‐10, increased with protein dose in rCsTegu21.6‐treated mBMDCs. (A) mBMDCs were cultured for 8 days and then stimulated with 1, 5, or 10 µg/mL rCsTegu21.6 for 18 h. Harvested cells were permeabilized and stained with PE‐conjugated anti‐mouse IFN‐γ (negative control), IL‐10, or IL‐12 antibodies after labeling with FITC‐conjugated anti‐mouse CD11c antibody. Fluorescence intensity was measured by flow cytometry. (B) Statistical results of the surface marker expression on mBMDCs. The results are shown as the mean ± standard error (SEM) and are representative of three independent experiments. **P < 0.01 compared to the PBS‐treated control group.

We also tested effects of rCsTegu21.6 on cytokine secretion and intracellular cytokine production in the mBMDCs. Following treatment with the recombinant protein, the mBMDCs secreted the pro‐inflammatory cytokines IL‐6 (*P* < 0.005), IL‐1β (*P* < 0.05), and TNF‐α (*P* < 0.05) in a dose‐dependent manner (Figure 3). IL‐10 (52 ± 0.6 pg/mL, *P* < 0.005) and IL‐12p70 (31.9 ± 1.1 pg/mL, *P* < 0.005) levels also moderately increased at the highest concentration (10 µg/mL) of rCsTegu21.6. Additionally, the intracellular IL‐12, as measured by flow cytometry, increased with rCsTegu21.6 protein concentration (*P* < 0.01), while that of IL‐10 was not statistically different (Figure 4). IFN‐γ was used as the negative control.

### Cell proliferation induced by rCsTegu21.6‐treated mBMDCs

**Figure 5:**
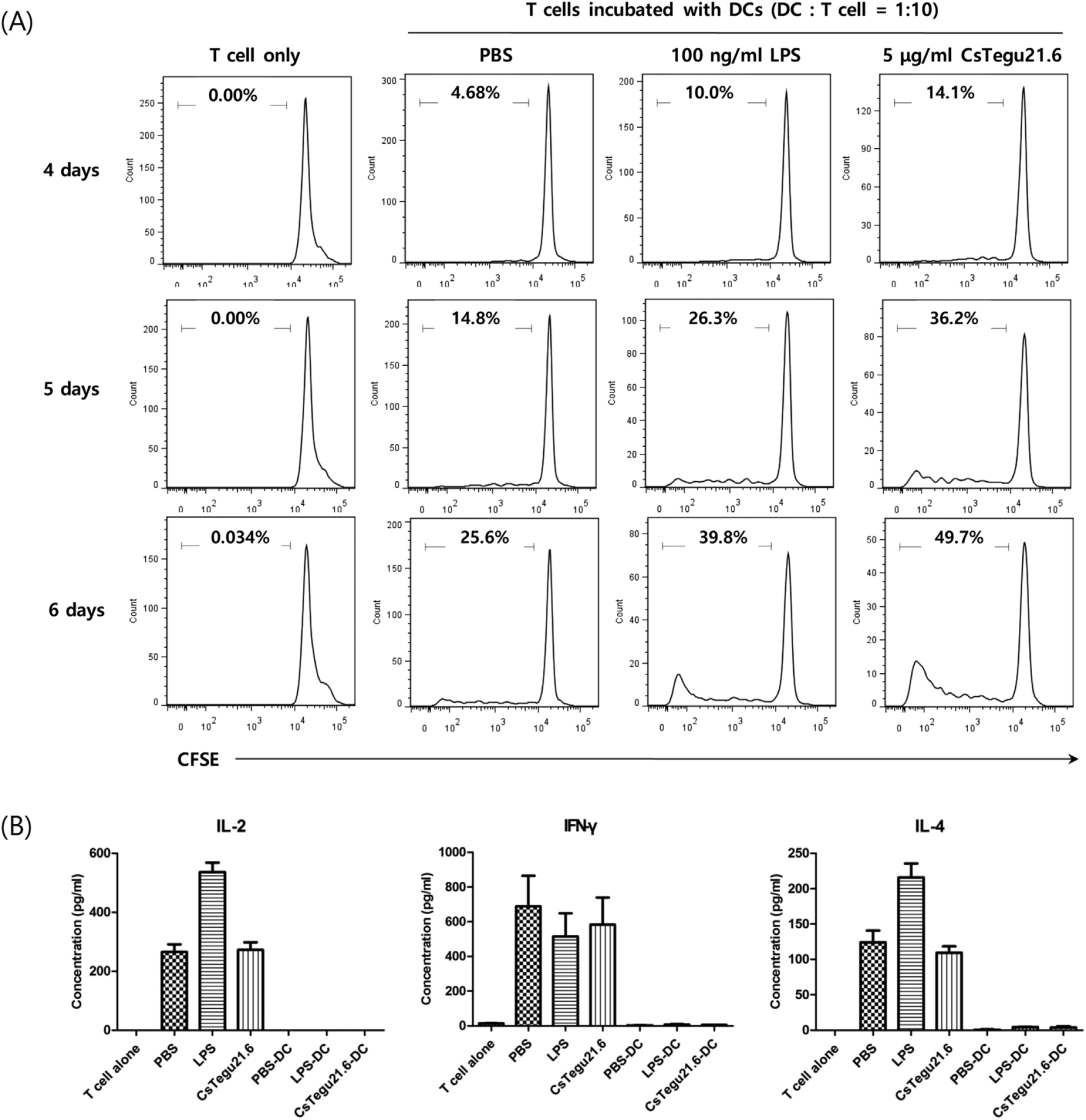
Proliferation of allogenic T cells, co‐cultured with rCsTegu21.6‐activated mBMDCs, increases over time. However, cytokines were not secreted from T cells by rCsTegu21.6 protein. mBMDCs were stimulated with 5 µg/mL rCsTegu21.6 for 18 h. Harvested mBMDCs (1 × 10^4^ cells/mL) were co‐incubated at a ratio of 1:10 with CFSE‐conjugated T cells derived from BALB/c mice. Co‐cultured cells were harvested, washed with DPBS, and analyzed at the time shown. (A) Flow cytometry analysis indicates that T cell proliferation increases over time in the co‐cultured cells. The *x*‐axis shows the fluorescence intensity, and the *y*‐axis depicts the number of cells. (B) Cytokine secretion from the T cells co‐cultured with CsTegu21.6‐treated mBMDCs was detected by ELISA. Statistical analyses were performed using two‐tailed *t* tests, and the results are shown as the mean ± standard error (SEM).

We investigated the activation of T cells by rCsTegu21.6‐treated mBMDCs via analysis of T cell proliferation and cytokine secretion. To this end, rCsTegu21.6 protein‐treated mBMDCs were co‐incubated with T cells isolated from a BALB/c mouse spleen. The activated mBMDCs increased the number of T cells over time (Figure 5A). However, the cytokine levels secreted from the T cells co‐incubated with treated‐mBMDCs were unchanged (*P* > 0.05) relative to the cells co‐incubated with PBS‐treated mBMDCs (Figure 5B).

### Specific IgG and isotype production in immunized mouse sera

**Figure 6:**
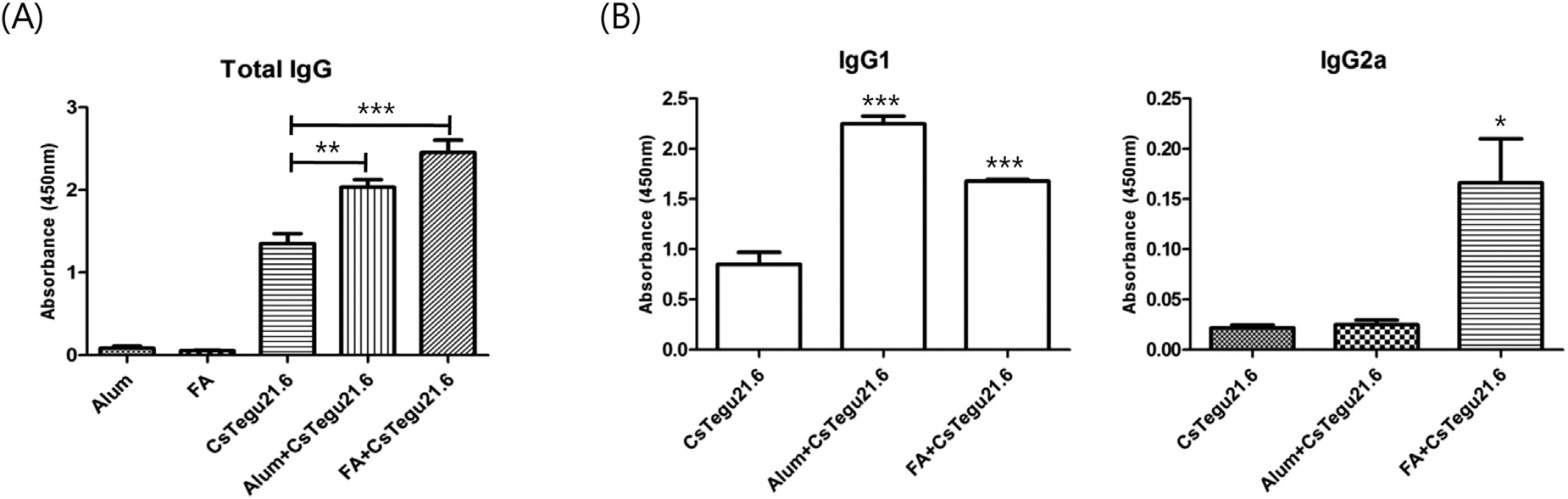
rCsTegu21.6 induced specific antibody production in C57BL/6 mice. rCsTegu21.6 (with or without alum adjuvant (Alum) or Freund's adjuvant (FA) was used to immunize C57BL/6 mice (*n* = 5 per group) three times after two‐week intervals. Total IgG (A) and their isotypes (B) were detected in the immunized serum by ELISA. The results are shown as the mean ± standard error (SEM). *P < 0.05, **P < 0.01, ***P < 0.001 compared to the rCsTegu21.6 only immunization group. IgG1 appears dominant in all groups.

rCsTegu21.6 protein provoked markedly higher production of specific total IgG regardless of the adjuvant used (*P* < 0.005) compared with that in the control groups as well as the protein only group (Figure 6A). As shown in Figure 6B, the IgG1 levels in both the alum and Freund's adjuvant‐mixed rCsTegu21.6 group were more elevated than that found for the protein only group (*P* < 0.0001), while the level of IgG2a was only slightly increased in the FA‐mixed group (*P* < 0.05). Thus, the IgG1 isotype appears to be dominant in all three groups. These results indicate that rCsTegu21.6 protein polarizes the murine immune response toward the Th2‐type regardless of the existence or type of adjuvant used.

### Cellular immune responses by rCsTegu21.6 in mice

**Figure 7:**
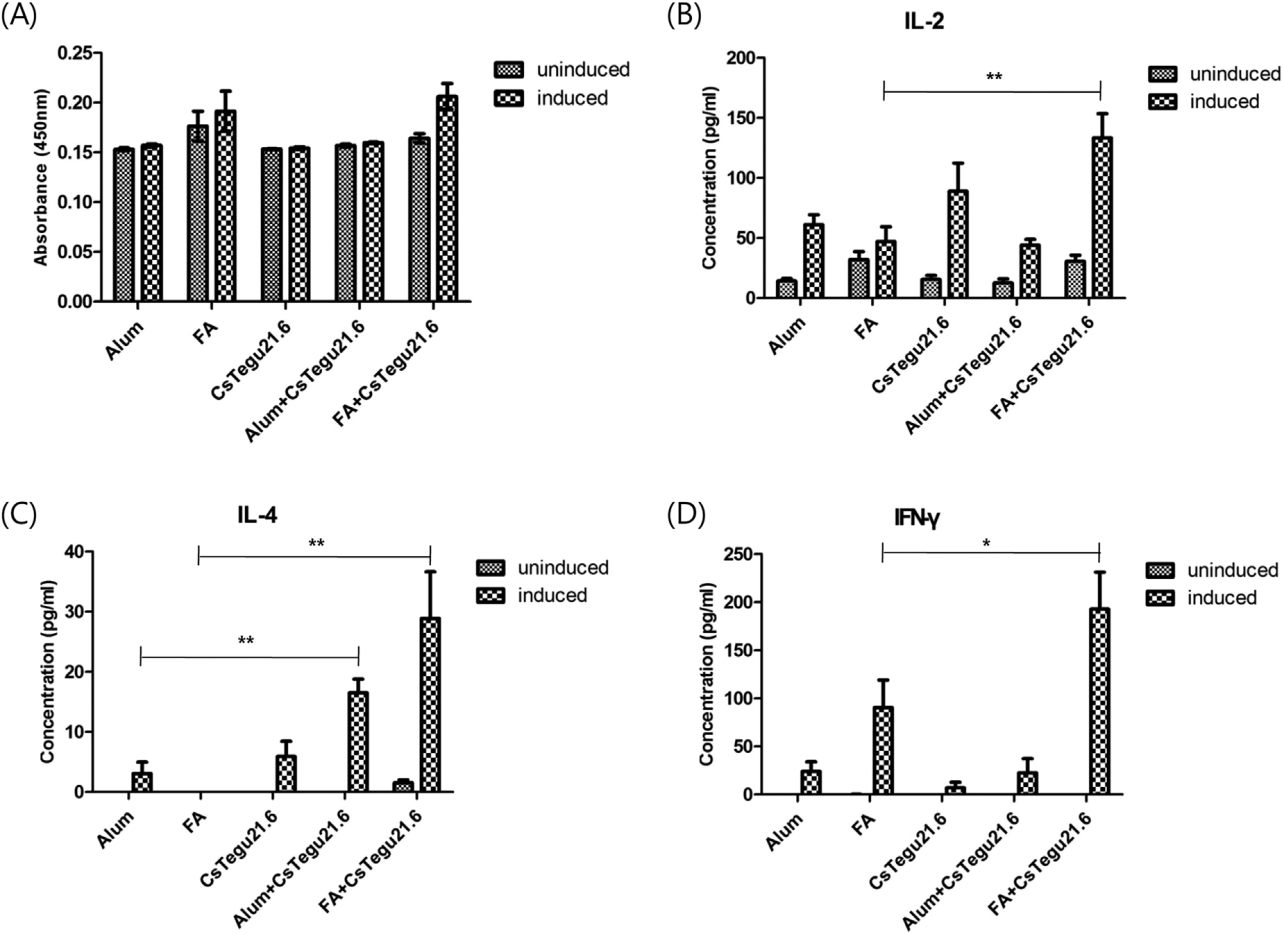
Cytokine secretion, but not the number of T cells, was increased in rCsTegu21.6‐immunized mice. T cells were isolated from rCsTegu21.6‐immunized mice and cultured either in the absence (uninduced) or presence of 10 µg/mL of rCsTegu21.6 (induced) for 70 h. (A) T cell proliferation was measured using a WST‐1 assay. (B–D) Cytokine levels were determined in the supernatants of harvested cells by ELISA. The results are shown as the mean ± standard error (SEM). *P < 0.05, **P < 0.01 compared to the adjuvant only immunization groups. rCsTegu21.6 protein mixed with FA activates IL‐2, IFN‐γ, and IL‐4 secretion, but alum‐mixed rCsTegu21.6 protein induced only an IL‐4 secretion.

To compare the cellular immune responses induced by rCsTegu21.6 protein, we investigated the responses of mouse splenocytes following immunization with rCsTegu21.6 emulsified with or without adjuvant. These data indicate that the number of splenocytes is not increased, regardless of rCsTegu21.6 immunization (Figure 7A). However, the level of cytokine secretion was significantly affected, with higher levels of IL‐2 (135.8 ± 51.7 pg/mL, *P* < 0.01), IL‐4 (30.7 ± 16 pg/mL, *P* < 0.01), and IFN‐γ (208.4 ± 83.9 pg/mL, *P* < 0.05) being secreted by splenocytes after immunization with rCsTegu21.6 mixed with FA compared to that in the adjuvant only group (Figure 7B–D). IL‐4 secretion of splenocyte was also increased (17.2 ± 6.5 pg/mL, *P* < 0.005) following immunization with rCsTegu21.6 mixed with alum adjuvant compared to that in the adjuvant only group, while secretion of IL‐2 and IFN‐γ were not significantly altered (*P* > 0.05). Taken together, these results confirm our earlier findings that rCsTegu21.6 protein immunization activates both the Th1 (IL‐2 and IFN‐γ) and Th2 (IL‐4) immune responses when mixed with FA, whereas alum adjuvant only stimulates Th2 cytokine production.

## Discussion

To date, there is currently no vaccine against *C. sinensis* infection, and only four tegumental proteins have been investigated for their use in vaccine development. In the present study, we carried out the first analysis of CsTegu21.6 as a potential vaccine target. To do so, the humoral and cell‐mediated immune responses to the recombinant protein (mixed with two different adjuvants) were evaluated using both in vitro experiments and immunization trials in mice.

Notably, the investigation of *C. sinensis* vaccine targets was initiated with an unpublished preliminary study investigating the innate immune responses mediated by four novel tegumental proteins: 20.8 kDa Cs‐tegumental protein (accession No. ABC47326), 20.6 kDa Cs‐tegumental protein (accession No. GAA49981.1), Cs‐tegumental calcium binding EF‐handed protein (accession No. ABZ82044.1) and CsTegu21.6 protein. This previous study performed in dendritic cells derived from C57BL/6 mice. Of these, only CsTegu21.6 shown to activate the dendritic cells, while the others showed no immunogenic properties (data not shown). These data not only focused our efforts on CsTegu21.6, but they also demonstrated that not all tegumental proteins are immunogenic.

Here, we initially demonstrated the localization of CsTegu21.6 protein in the worm by immunohistochemistry, using mouse poly‐sera against the rCsTegu21.6 protein. Not surprisingly, CsTegu21.6 proteins were abundantly located in the tegument of adult *C. sinensis* worms, indicating that it could directly respond to the host's immune system following infection. Furthermore, direct activation of murine immune cells by rCsTegu21.6 protein was confirmed in vitro using isolated mBMDCs. The presence of rCsTegu21.6 protein accelerated the expression of co‐stimulatory molecules CD40 (77%), CD80 (52%), and CD86 (46%) on dendritic cells. These molecules often used as activation indicators of T cell‐regulated immune responses. Secretion of the pro‐inflammatory cytokines (IL‐6, IL‐1β, and TNF‐α) also increased in a dose‐dependent manner, further confirming that the dendritic cells are indeed activated by rCsTegu21.6 protein. IL‐10, IL‐12p70, and intracellular IL‐12 concentrations were also increased and correlated with the concentration of rCsTegu21.6 protein, but intracellular IL‐10 was not produced. It thought that IL‐10 produced in the cells during protein treatment was mostly secreted, so intra‐cellular IL‐10 remained at the baseline level. The downstream effects of these rCsTegu21.6‐activated mBMDCs on immune function also demonstrated by co‐culturing these activated dendritic cells with isolated T cells. Interestingly, the proliferation of these co‐cultured T cell was time‐dependently increased, but there was no effect on the concentration of secreted cytokines. These results indicate that CsTegu21.6 could stimulate dendritic cell‐mediated T cell proliferation. Moreover, this protein alone is not sufficient to induce a change in cytokine secretion. As a result, it seems that rCsTegu21.6 protein alone does not induce a strong cellular immune response, and that it is necessary to elicit a stronger immune response along with the adjuvant.

Subsequent to this in vitro analysis, we also confirmed the humoral and cell‐mediated immune responses following rCsTegu21.6 protein immunization in mice. Total IgG against rCsTegu21.6 protein produced in all groups except the PBS control group, but their titer varied depending on the type of adjuvant used. Antibody production was the most effective when FA was used. We further analyzed the IgG subtype in these test groups, and it appears that using alum adjuvant induced higher IgG1 levels than IgG2a, whereas using FA lowered the IgG1/IgG2a ratio compared to using alum adjuvant. This is supported by the result from another study 28. Furthermore, rCsTegu21.6 protein stimulated not only overall antibody production, but also cytokine secretion from splenocytes. The alum adjuvant‐mixed rCsTegu21.6 group induced only IL‐4 secretion, while IL‐2, IL‐4, and IFN‐γ were all significantly altered in the FA‐mixed protein group. These results indicate that mixing the rCsTegu21.6 protein with FA stimulated both Th1 and Th2 immune responses and appears to be more effective overall, while protein mixed with alum adjuvant induced a less effective response that was predominantly Th2. This is not the first time that discrepancies have been observed in immunogenicity of a single protein. Indeed, Maggioli et al. 29 report that protein‐mediated immune responses and protective effects are largely dependent on the type of adjuvant used. Multiple studies have indicated that FA may be too toxic because of its origin (made using killed Mycobacterium 30), and its use has been associated with severe pathological changes regardless of the injection route 31. For these reasons, rCsTegu21.6 mixed with FA may not be the best inducer of immune responses actually (vs. alum adjuvant‐mixed protein or recombinant protein alone).

It is important to note that vaccines against rCsTegu21.6 or any other tegumental protein may not elicit a full immune response when used alone. Liver flukes are large, multi‐cellular parasites that likely use multiple pathways to avoid or circumvent the host immune system. Thus, it may be more beneficial to utilize a combination of two or more vaccine candidate antigens to develop an effective vaccine. In fact, several vaccine studies performed for other parasites have revealed that multi‐vaccines made by combining well‐known vaccine candidate antigens were more effective than a single antigen used alone 32, 33. While rCsTegu21.6 appears to be an ideal candidate for vaccine development, additional studies investigating the combinatorial effect of immunogenic proteins from *C. sinensis* is warranted to determine the effects of multi‐target vaccines to prevent *C. sinensis* infection 34, 35, 36.

In this study, a recombinant form of 21.5 kDa tegumental protein of *C. sinensis* (rCsTegu21.6) has been demonstrated to be a novel vaccine candidate owing to its immune‐dominant reactivity in both innate and adaptive immune responses. These immunogenic responses were shown both in vitro and in vivo, whereby rCsTegu21.6 protein not only induced surface molecule expression and cytokine secretion in mBMDC, but also induced T cells proliferation when co‐cultured with mBMDCs activated by rCsTegu21.6 protein. rCsTegu21.6 protein also induced antibody production and cytokine secretion from splenocytes. While rCsTegu21.6 protein more strongly activated humoral and cell‐mediate immune response when mixed with FA compared to that with alum adjuvant and adjuvant‐free mixes, caution should be used when considering the application of FA in later clinical applications because of its toxicity. To our knowledge, this is the first study to demonstrate the viability of rCsTegu21.6 as a vaccine candidate. Additional studies are needed to examine the protective effects of rCsTegu21.6 vaccines, including single and multi‐antigen vaccines, against *C. sinensis* infection and to elucidate the underlying host defense mechanism. These results serve as a foundation of basic data for the development of clonorchiasis vaccines based on the tegumental proteins of *C. sinensis* adult worms.

## Ethics Approval and Consent to Participate

All animal experiments were approved by the Committee on the Ethics of Animal Experiments of the Korean Centers for Disease Control & Prevention (permit number KCDC‐171‐14‐2A). Experimental animals were maintained and handled in strict accordance with institutional guidelines of the Committee on the Ethics of Animal Experiments of the Korean Centers for Disease Control & Prevention.

## Authors’ Contributions

EJC and JWJ designed the study. EJC performed the experiments, analyzed the data, and drafted the manuscript. MRL and YJK did the protein analysis. SHC performed the study design and manuscript revision. All authors have read and approved the final manuscript.

## Conflict of Interest

None declared.
